# Distinct resting state neural activity in chronic pain patients who respond to transcranial electric stimulation for pain relief

**DOI:** 10.3389/fnhum.2025.1546414

**Published:** 2025-07-15

**Authors:** Alba Fernández, Lara Rubal-Otero, Antonio Gil-Ugidos, Diego Pinal, Alberto Jacobo González-Villar, María Teresa Carrillo-de-la-Peña

**Affiliations:** ^1^Brain and Pain (BaP) Lab, Department of Clinical Psychology and Psychobiology, University of Santiago de Compostela (USC), Santiago de Compostela, Spain; ^2^Institute of Psychology (IPsiUS), Universidade de Santiago de Compostela, Santiago de Compostela, Spain; ^3^Foundation for Health Research Institute of Santiago de Compostela (FIDIS), Santiago de Compostela, Spain; ^4^Psychological Neuroscience Lab, Department of Basic Psychology, School of Psychology, Research Centre in Psychology, University of Minho, Braga, Portugal

**Keywords:** chronic pain, transcranial direct current stimulation (tDCS), transcranial alternating current stimulation (tACS), electroencephalography, power spectral density (PSD)

## Abstract

**Introduction:**

Transcranial electric stimulation (tES) techniques have shown promising results for pain relief in chronic pain. However, little is known about the mechanisms by which these non-invasive neuromodulation techniques produce analgesic effects. Besides, previous studies underscore the need to identify profiles of patients with a better response to tES.

**Methods:**

In this randomized clinical trial (ClinicalTrials.gov: NCT05099406), we studied whether tES modulate brain oscillatory activity by recording resting state EEG (eyes open) from 106 chronic pain patients before and after a 15-day home-based intervention with either transcranial direct or alternate current stimulation, or sham stimulation. Power spectral density (PSD) at rest was analyzed in the theta, alpha, and beta frequency bands, in two 2 × 2 designs with the factor time (pre vs. post intervention session) and group, with each active tES condition being compared against the sham stimulation. Additionally, we compared responders vs non-responders to active tES (according to their reported pain relief after the intervention) in the same PSD indices.

**Results:**

The analysis showed that the intervention had no significant effects on PSD in any band, and thus revealed that understanding the physiological mechanisms of the analgesic effects of tES remains a pending matter. However, higher PSD in the theta band was observed for responders compared to non-responders regardless of the assessment session.

**Discussion:**

This finding suggests that the theta-band oscillatory activity of patients with chronic pain could be a promising prognostic biomarker for the effectiveness of tES and opens a new avenue for individualizing tES interventions.

## 1 Introduction

Chronic pain is a widespread health problem with a great negative impact on the quality of life of its sufferers. It has been estimated that about 19–25% of adults in Europe suffer from chronic pain of moderate to severe intensity, with increased prevalence as the population ages (Breivik et al., 2006; Leadley et al., 2012). Given its varied etiology, the causal mechanisms of chronic pain are not yet clearly understood, which in turn impacts its possible treatments. In fact, managing and relieving chronic pain remains still a challenge for healthcare providers. Common treatments for chronic pain are mostly pharmacological, yet for as many as 40–60% of chronic pain patients this line of treatment does not provide meaningful pain relief (Dworkin et al., 2007).

In recent years, neuromodulation approaches, such as non-invasive transcranial electrical stimulation (tES), have been studied as a possible treatment for pain relief in chronic pain patients. Among these techniques, transcranial direct current stimulation (tDCS) and transcranial alternate current stimulation (tACS) have shown promising results in the management of pain for chronic pain patients (Ahn et al., 2019; Fregni et al., 2021). Both tDCS and tACS are non-invasive techniques that modulate cortical excitability using a low-intensity current. In the case of tDCS, a weak current is applied constantly between the electrodes placed on the scalp (usually 2). This has been shown to modulate transmembrane potential, either increasing or decreasing it depending on the polarity of the stimulation, thus changing neural excitability (Nitsche et al., 2008; Nitsche and Paulus, 2000). Regarding pain relief, anodal tDCS over M1 has currently a level B of evidence (i.e. probably effective) to reduce chronic pain in fibromyalgia, neuropathic pain, migraines, and postoperative pain (Fregni et al., 2021). In the case of tACS, a small alternating current at a specific frequency is applied between two electrodes placed on the scalp. This has been seen to affect the entrainment and modulation of brain oscillatory activity (Fröhlich et al., 2014; Helfrich et al., 2014). Despite tACS not being as studied for chronic pain as tDCS there have been some promising results managing chronic pain with this technique, although the evidence is conflicting (Chang et al., 2023).

The strongest rationale underlying the use of these techniques to treat chronic pain is that, by targeting pain-related cortical activity, they might influence pain processing or modulation (Jensen et al., 2014; O'Connell et al., 2018; Pinto et al., 2018). Indeed, differences in cortical activity have been observed in patients with chronic pain of diverse etiologies compared to healthy populations (see Pinheiro et al., 2016 for a review). In this regard, increased delta activity has been found in chronic pain patients compared to healthy population (Krupina et al., 2020; Lopes et al., 2024) as well as a reduction in fibromyalgia patients (González-Roldán et al., 2016). Regarding theta activity, while some studies have reported an increase in theta (4–8 Hz) oscillatory activity at rest in patients with neuropathic pain (Sarnthein et al., 2006; Stern et al., 2006), others have reported its reduction in patients with fibromyalgia (Hargrove et al., 2010; Navarro López et al., 2015; see Silva-Passadouro et al., 2024 for a review). In the same way, both increases (Bell et al., 2001; Fauchon et al., 2022; Prichep et al., 2018; Vanneste and De Ridder, 2021) and reductions (Hargrove et al., 2010; Navarro López et al., 2015; Vanneste et al., 2017; Villafaina et al., 2019) of alpha (8–12 Hz) activity at rest have been reported for different chronic pain conditions compared to healthy controls. Conversely, the reports of differences in beta (13–30 Hz) activity at rest seem less divided, with most studies reporting increased activity in various chronic pain conditions, especially in frontal regions (De Ridder and Vanneste, 2017; González-Roldán et al., 2016; Hargrove et al., 2010; Lim et al., 2016; Makowka et al., 2023). Furthermore, regarding the gamma frequency band, although activity in this frequency has been associated with nociception and tonic pain (see Ploner et al., 2017 for a review), only a few studies have found an increase of gamma activity in chronic pain patients compared to healthy population (Michels et al., 2010; Vanneste et al., 2018; Zhou et al., 2018).

Adding to this complex landscape, there is little research about how tES modulates the cortical activity of patients with chronic pain when used as a pain relief treatment. To the best of our knowledge, only four studies have measured the changes in cortical oscillatory activity in chronic pain patients after treatment with either tDCS or tACS, with varying results. Thus, regarding tDCS, only changes in slow frequencies have been observed, with one study finding an increase in theta and low alpha (8–10 Hz) frequencies after one session of tDCS in patients with chronic visceral pain (Thibaut et al., 2017), and a study finding a decrease spectral power in the faster alpha frequencies (alpha-2), in parietal and frontal regions in fibromyalgia patients after five sessions of tDCS (De Melo et al., 2020). At the same time, regarding tACS, one study found an increase in alpha activity after one alpha-tACS session that correlated with pain relief in patients with chronic lower back pain (Ahn et al., 2019), while another study found an increase in alpha-1 (8–10 Hz) activity in fibromyalgia patients after one session of beta-tACS (Bernardi et al., 2021). As it can be seen, there is a gap in this field, with few studies performed and a lack of consensus about the effects of tDCS and tACS in cortical activity at rest in the chronic pain population. Moreover, except for one study, brain activity was measured after one session of tES; however, the positive effects of tDCS on chronic pain are mainly observed after at least five stimulation sessions (Fregni et al., 2021). To the best of our knowledge, changes in cortical activity following a tES treatment for chronic pain have not been studied in the delta and gamma frequency bands.

Additionally, there is evidence for changes in functional connectivity in different frequency bands when comparing chronic pain patients with healthy population (Kim and Davis, 2021). Increases in theta connectivity have been reported; however, decreases have been also observed in global connectivity (Choe et al., 2018) and between the insula and the default mode network (Hsiao et al., 2017). A recent study found a correlation in global connectivity in the theta frequency band of chronic pain patients with decreases in pain intensity and pain related disability after interdisciplinary multimodal pain therapy (Heitmann et al., 2022). Changes in functional connectivity in other frequency bands have also been observed, with decreased connectivity in the alpha band in neuropathic pain patients compared to healthy controls (Vanneste and De Ridder, 2021), as well as increases in beta connectivity (González-Roldán et al., 2016; González-Villar et al., 2020) in fibromyalgia patients compared to healthy populations.

Previous literature on the effect of neurostimulation in chronic pain is mainly based on analysis at a group level, although there is great inter-individual variability in the response to these interventions. Ciampi de Andrade and García-Larrea (2023) underscore the need to individualize therapeutic transcranial neuromodulation for chronic pain. As has been suggested in previous literature (Thorp et al., 2018), functional changes in brain activity of patients with chronic pain have the potential of predicting individual responses to specific therapeutic interventions and thus serve as a gateway for personalized medicine. Nevertheless, resting state EEG differences between responder vs. non-responder patients to tES have not been investigated so far.

As a response to the above gaps, in the present study, we aimed to improve the understanding of the neural mechanisms underlying the analgesic effects of tES. To that end, more than one hundred patients diagnosed with different chronic pain conditions undertook either a tDCS or alpha-tACS home-based intervention for pain relief for 15 consecutive days. Our first objective was to study how tES modulates brain activity. Electroencephalographic (EEG) activity at rest was recorded before and after treatment; and cortical oscillatory activity was analyzed in the delta, theta, alpha, beta and gamma frequency bands. As altered EEG activity at rest in these bands has been described in chronic pain patients, it was expected that tES would modulate activity in these bands. Moreover, our second objective was to characterize the brain activity, at the aforementioned frequency bands, of patients who reported pain relief to active tES intervention compared to patients who had no positive response to the intervention. Finally, functional connectivity was also studied in responders and non-responders in order to characterize their global connectivity before and after tES treatment.

## 2 Material and methods

### 2.1 Participants

The sample was comprised of 106 patients diagnosed with a chronic pain condition (84 women/22 men; mean ± SD age = 49.75 ± 8.34 years). The eligibility of participants was determined by telephone screening before the first visit. Inclusion criteria were: (1) age between 25 and 65 years old; (2) being diagnosed with any chronic pain condition by a physician; (3) mean pain intensity for the last 3 months scored as ≥4/10 on a Numerical Rating Scale (NRS)^1^; (4) mean pain intensity during the last week scored as ≥5/10 on a NRS scale; and (5) stable pharmacological treatment for over 2 months (except pain medication that could vary depending on patient status). Exclusion criteria were: (1) presence of pain associated with a cancer diagnosis; (2) ongoing or planned pregnancy; (3) history of drug abuse; (4) unstable medical conditions; (5) implanted intracranial devices or stimulators; (6) history of neurosurgery; (7) traumatic brain injury with loss of consciousness or cortical lesions; (8) family history of epilepsy or active epilepsy; and (9) suffering any psychiatric disorders (other than depression and anxiety).

Participants were recruited in collaboration with the Pain Management Centers of the University Hospital of Santiago de Compostela (CHUS) and University Hospital of Vigo (CHUVI) (Spain), where patients diagnosed with refractory, pharmaco-resistant chronic pain that met the criteria to participate were informed about the study. Researchers contacted the patients who expressed interest in participating in the study and who gave consent to be contacted by phone. Additionally, patients who became aware of the study through other channels (e.g., social media, local press…) and approached the researchers were also included in the study if they met the criteria for participation.

All participants gave their informed consent before the start of the study. The study complied with the Declaration of Helsinki and was approved by the Research Ethics Committee of Santiago-Lugo (code 2021/021). The study was preregistered at Clinicaltrials.gov (ID number: NCT05099406).

### 2.2 Study design

The present study is a randomized, double-blind, sham-controlled clinical trial that was conducted at the University of Santiago de Compostela (USC) and the Pain Management Center of the University Hospital of Vigo (CHUVI).

Participants were randomly assigned to three treatment groups (active tACS, active tDCS and sham tES) by an external researcher to the project. An allocation ratio of 2:2:1 was used, resulting in 43 participants in the active tDCS group (31 women/12 men; mean ± SD age = 49.58 ± 7.86 years), 43 participants in the active tACS group (36 women/7 men; mean ± SD age = 49.9 ± 8.54 years), and 20 participants in the sham tES group (17 women/3 men; mean ± SD age = 49.75 ± 9.29 years).

The study comprised three distinct phases: a pre-treatment period, a tES treatment period, and a post-treatment period. Each phase lasted 15 days and had an in-person visit at its start. After the first in-person visit (pre-treatment), patients were asked to provide daily reports on their pain intensity and symptoms for 15 days by answering online NRS. In the second in-person visit (start of treatment), resting state EEG with eyes open (rsEEG) was recorded for 6 min; and afterwards, patients were provided instructions on how to use the tES device and completed the first tES session under the supervision of the researchers. Patients were advised to always perform the stimulation session at the same time of the day, when they could be relaxed and carefree. This time could be different for each patient, as each had different obligations and schedules.

Participants took home the stimulation device, a headband with sponge electrodes fixed in place, and the necessary material to complete the remaining 14 tES sessions at their own home. To complete the tES sessions at home, researchers sent participants a daily code (via WhatsApp) that enabled them to carry out the tES treatment. Additionally, patients reported their daily symptoms, pain intensity, and side effects by filling out an online questionnaire after every tES session. After 15 days of tES treatment, rsEEG was again recorded for 6 min in the third in-person visit (post-treatment). After this visit, patients kept reporting their daily symptoms and pain intensity for another 15 days by filling out the online NRS.

### 2.3 Transcranial electric stimulation protocols

An external researcher randomly assigned the participants to one of three treatment groups: tDCS, tACS, or sham stimulation. All the treatment groups received 15 tES sessions.

Stimulation was administered by a Soterix device (Soterix 1 × 1 tES mini-CT) with two sponge electrodes (5 × 5 cm) for tDCS protocols (active and sham), and three sponge electrodes (5 × 5 cm) for tACS protocols (active and sham). The external researcher who assigned the participants to a treatment group, also assigned each participant a daily stimulation code. The researchers in charge of tES delivery were only given these codes to enter into the tES device and the patient's assigned protocol (tDCS or tACS). The Soterix device displays no information that could provide any insight into whether active or sham stimulation is applied. Thus, the researchers remained blind to the treatment condition.

The tES protocols had the following parameters:

***Transcranial direct current stimulation (tDCS):*** anodic tDCS was applied over the left primary motor cortex (M1), with the anode placed on the C3 electrode position in the International System 10–20 and the cathode on FP2. Stimulation intensity for each session was 2 mA delivered for 20 min with 15 s of ramp up and ramp down at the beginning and end. This montage was selected as it has the best evidence in patients with chronic pain (Fregni et al., 2021).

***Transcranial alternating current stimulation (tACS):*** alpha-tACS was applied over the dorsolateral prefrontal cortex (DLPFC) using two sponge electrodes in positions F3 and F4. Another sponge electrode was placed in the Pz position and used as a “return” electrode. Bipolar stimulation was applied with a sine wave at 10 Hz frequency and an intensity of 1 mA (zero-to-peak) for 20 min with 15 s of ramp-up and ramp-down. We used this montage to replicate previous findings by (Ahn et al., 2019).

***Sham stimulation:*** sham stimulation was used as a placebo treatment in order to configure a control group. Half of the sham stimulation group participants were assigned the tDCS montage, while the other half were assigned the tACS montage. The protocol for sham stimulation was identical to that of the corresponding active group, except that current was only delivered during the ramp-up and ramp-down moments. To ensure the success of the blinding procedure, participants were asked to report whether they believed they received active or sham stimulation after the treatment was finalized. Data from both sham groups were aggregated for the analysis.

To ensure the success of the blinding procedure, participants were asked to report whether they believed they received active or sham stimulation after the treatment was finalized.

**Figure 1:**
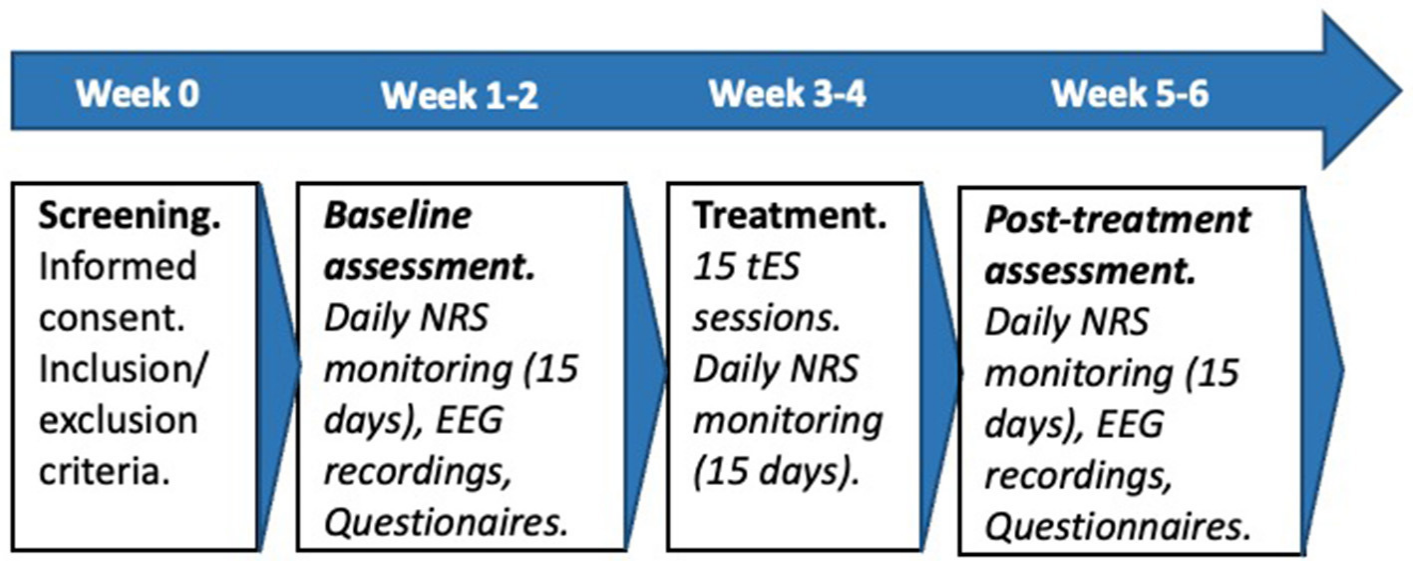
Flowchart of the procedure. NRS, numeric rating scales; tES, transcranial electric stimulation; EEG, electroencephalography.

The flowchart of the procedure is depicted in Figure 1.

### 2.4 Resting state EEG recording

As explained above, rsEEG was recorded for 6 min at two time points (pre- and post-treatment). During the recording, participants sat comfortably inside a Faraday chamber with attenuated light and noise. Participants were asked to look at a fixed point in front of them, avoid excessive body and eye movements, and keep their eyes open while staying relaxed. The EEG recording session additionally included the completion of a sensory perception and a cognitive task (55 min total duration). Results from such tasks are out of the scope of the present work.

Resting state EEG activity was recorded using 62 active electrodes inserted in an electrode cap (ActiChamp system; Brain Products Inc.). The electrodes were placed according to the 10-10 International System, with nose tip reference and the ground electrode located in FPz. Horizontal eye movements were registered using two additional surface electrodes on the outer canthi of both eyes; and vertical eye movements were recorded using an active electrode placed 2 cm below the right eye.

The EEG signal was recorded with an online bandpass filter between 0.1 and 100 Hz, with a 50 Hz notch filter and digitized at 500 Hz. Electrode impedances were kept below 10 KΩ.

### 2.5 Resting state EEG processing

EEG data was preprocessed and analyzed using MATLAB (R2023b) and the toolbox EEGLab (v2023.1) (Delorme and Makeig, 2004). Data was band-pass filtered between 0.5 and 80 Hz using a finite impulse response (FIR) filter. The *clean raw data* plugin (Kothe et al., 2019) was used for the automated detection and removal of channels with a flat signal or with low correlation (*r* ≤ 0.75) with neighboring channels. Following this step, data was re-referenced to an average reference and artifact subspace reconstruction (ASR), as implemented in the *clean raw data* plugin, was used to correct large transient artifacts. Next, an independent component analysis (ICA) decomposition was conducted, followed by the removal of the estimated independent components showing ocular or muscular artifacts, or with a low probability of containing brain activity ( ≤ 20%). The plugin IC Label was used to inspect these independent components (Pion-Tonachini et al., 2019). EEGLab was used to reconstruct the EEG signal without the artifactual components. After this, the continuous signal was partitioned into 2 second segments, which were then marked as containing artifacts and automatically removed from the data if they met any of these criteria: (1) presence of values of ±100 μV; (2) presence of trends with slopes exceeding 75 μV; (3) presence of improbable data or abnormal distribution with a single channel limit of 5 SDs; and (4) spectral power outside the range of ±50 dB for frequencies between 0 and 2 Hz, and/or outside the range of ±100 dB for frequencies between 20 and 40 Hz. Power spectral density (PSD) was computed for all electrodes at the sensor level using the EEGLab “*spectopo*” function, which computes PSD in a given frequency range. PSD was thus computed for the following frequency bands: delta (2–4Hz), theta (4–7.5 Hz), alpha (8–12 Hz), beta (13–30 Hz) and gamma (30–45 Hz). For each participant and frequency band, the mean PSD value was computed using the EEGLab function “*std_spec*”. Mean PSD values were subsequently used for statistical analysis.

The weighted phase lag index (wPLI) (Vinck et al., 2011) was computed from 2 to 40 Hz in 1 Hz steps. The wPLI measures the asymmetry of the distribution of phase differences between two signals, where each phase difference is weighted by the magnitude of its imaginary component, returning values between 0 (no phase-locking or phase-locking with zero lag) and 1 (perfect non-zero-lagged phase synchronization). The mean wPLI values among all electrode pairwise combinations were computed, then the mean values of each frequency band of interest were computed for group comparisons.

### 2.6 Statistical analysis

All statistical analyses were performed in MATLAB (R2023b) using the EEGLab STUDY utilities. Connectivity analyses were performed in the statistical software JAMOVI.

The first statistical analysis was performed to explore the effects of tES treatment on the mean PSD of participants in the delta, theta, alpha, beta and gamma frequency bands. To that end, tests were run using a set of 2 × 2 designs with factors time (pre- and post-treatment sessions) and group. As there were three groups, the design was used to compare active tDCS vs. sham stimulation and active tACS vs. sham stimulation. For all designs, non-parametric permutation statistics with 5,000 randomizations were computed with an alpha level at *p* ≤ 0.05. Multiple comparisons were corrected by applying the false discovery rate (FDR) correction (Benjamini and Yekutieli, 2001).

The second statistical analysis was performed in order to compare the mean PSD of participants who reported pain relief after active tES treatment to those who did not experience significant pain relief. To this end, we classified the participants in the active tDCS and tACS groups as “responders” or “non-responders” to the treatment, using the daily pain intensity NRS filled in the pre-treatment and post-treatment phases of the study. Participants were classified as “responders” when there was, at least, a 30% decrease in pain intensity comparing the average of the pre- and post-treatment NRS. Reductions in pain intensity of 13% have been described to reflect minimally important changes (Dworkin et al., 2008); thus, in the present study a 30% reduction was chosen to ensure that analyses would be focused on patients who obtained a meaningful benefit from the intervention, given that reductions in pain intensity.

After classifying participants into “responders” and “non-responders”, a 2 × 2 design (time x group) was used to compare the mean PSD of “responders” before and after treatment to that of “non-responders”, separately in the delta, theta, alpha, beta and gamma frequency bands. Non-parametric permutation statistics with 5,000 randomizations were computed with an alpha level at *p* ≤ 0.05, and FDR was applied to correct for multiple comparisons.

Finally, for the wPLI connectivity analysis, a 2 × 2 ANOVA was performed with factors “group” (responders vs. non-responders) and “time” (pre-treatment vs. post-treatment) for the theta, alpha and beta frequency bands. Analyses were performed with an alpha level at *p* ≤ 0.05.

## 3 Results

### 3.1 Descriptive data

Table 1 shows the mean pain intensity NRS data for 15-day in each period (pre-treatment, during-treatment and post-treatment). As may be seen, only the groups undergoing active tES (tDCS or tACS) reduced their pain ratings during and after treatment. The rate of patients with a positive response to treatment was 25.88%; they showed a large decrease of pain ratings specially at post-treatment.

**Table 1:** Numeric rating scales (NRS) for pain intensity.

	**Pre-treatment**		**During treatment**		**Post-treatment**	
	**Mean**	**SD**	**Mean**	**SD**	**Mean**	**SD**
**tDCS (*****n*** = **43)**	6.09	1.57	5.31	1.83	4.99	1.84
**tACS (*****n*** = **43)**	6.33	1.35	5.41	1.63	5.36	1.84
**Sham (*****n*** = **20)**	6.05	1.58	6.41	1.65	6.10	2.16
**Responders (*****n***= **22)**	5.83	1.63	5.20	1.72	2.87	1.08
**Non-responders (*****n*** = **63)**	6.35	1.39	5.42	1.74	5.97	1.29

Mean of 15 days in each period (pre-treatment, during and post-treatment), for each group of treatment.

### 3.2 Effects of tES on power spectral density

For the comparison between the active tDCS and sham stimulation groups, there were no significant differences in mean PSD in the delta, theta, alpha, beta and gamma frequency bands; neither between groups (tDCS vs. sham stimulation; *p* > 0.05 for all channels in the three frequency bands, FDR corrected) nor between time points (pre-treatment vs. post-treatment; *p* > 0.05 for all channels in the three frequency bands, FDR corrected). No significant interactions between these two factors were observed in any electrode for any of the analyzed frequency bands (*p* > 0.05 for all channels, FDR corrected).

Regarding the comparison between the active tACS and sham stimulation groups, no significant differences in mean PSD were observed between groups in the delta, theta, alpha, beta and gamma frequency bands (p > 0.05 for all channels in the three frequency bands, FDR corrected). Likewise, there were no significant differences in PSD between time points (pre-treatment vs. post-treatment; p > 0.05 for all channels in the three frequency bands, FDR corrected), nor significant interactions between group and time points in any electrode for the theta, alpha and beta frequency bands (p > 0.05 for all channels in the three frequency bands, FDR corrected).

### 3.3 Power spectral density of responders vs. non-responders to tes treatment

The “responders” group included 22 participants (18 women/4 men; mean ± SD age = 51.18 ± 7.85 years), while the “non-responders” group was formed by 64 participants (49 women/18 men; mean ± SD age = 49.27 ± 8.27). Of the 22 responders to tES intervention, 10 were part of the tACS group (45.5%) and 12 were part of the tDCS group (54.5%). A Chi Square test indicates that there was no significant difference between groups in the proportion of responders (χ^2^ = 0.185; *p* = 0.667).

**Figure 2:**
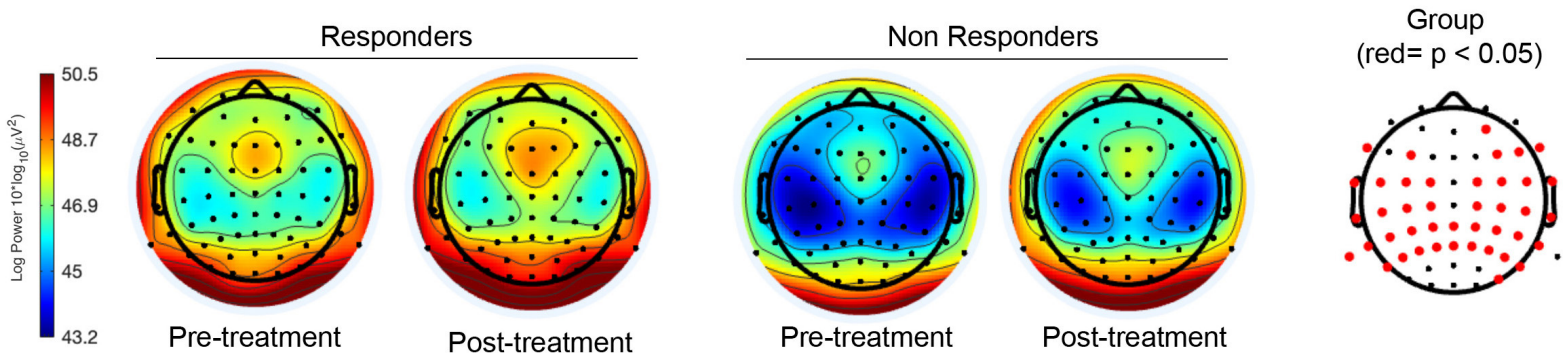
Topographical maps with the PSD of responders and non-responders to active tES in the theta band, before and after the intervention. The map on the right corresponds to a topographical representation of EEG channels with statistical significance for the “group” factor (*p* < 0.05, FDR corrected) marked in red. The figure on the left correspond to the topographical maps of both responders and non-responders before and after tES treatment, with blue color indicating lower theta activity and red color greater theta activity.

**Figure 3:**
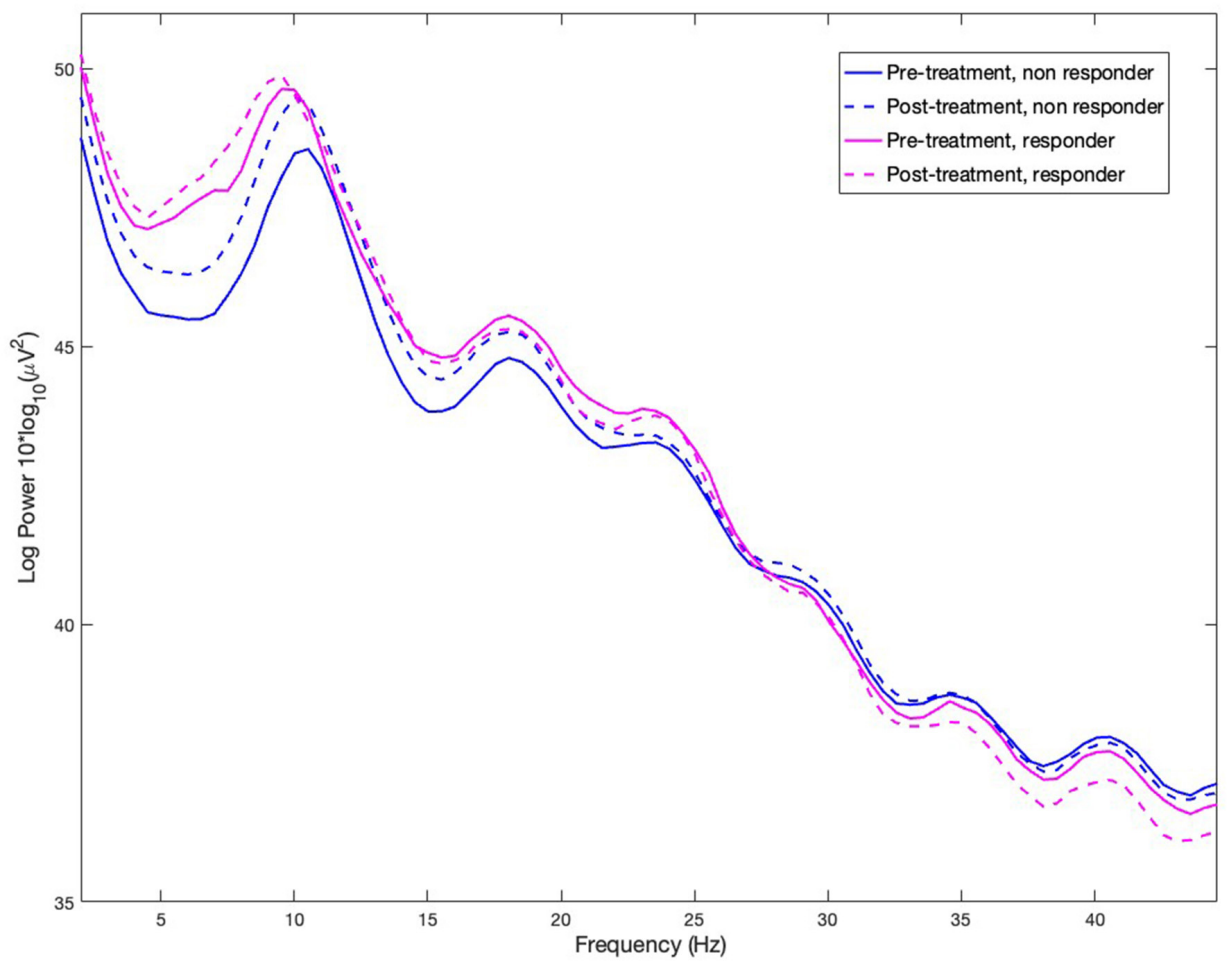
Power spectral density (PSD) of responders and non-responders to active tES in the frequency range from 2 to 45 Hz at pre-treatment and post-treatment time points.

We found statistically significant differences between groups in the theta frequency band (Figure 2). Significantly higher theta mean PSD was observed in responders compared to non-responders (Figure 3), predominantly in central, parietal and temporal electrodes (*p* < 0.05, FDR corrected). There were no significant differences between groups in the delta, alpha, beta and gamma frequency bands after applying FDR correction.

No significant differences were found between time points (pre-treatment vs. post-treatment) after applying FDR correction in any of the analyzed frequency bands, with greater theta activity observed in responders at both time points. No significant interactions were found between groups and time points in the analyzed frequency bands.

### 3.4 Connectivity of responders vs. non-responders to tES treatment

**Figure 4:**
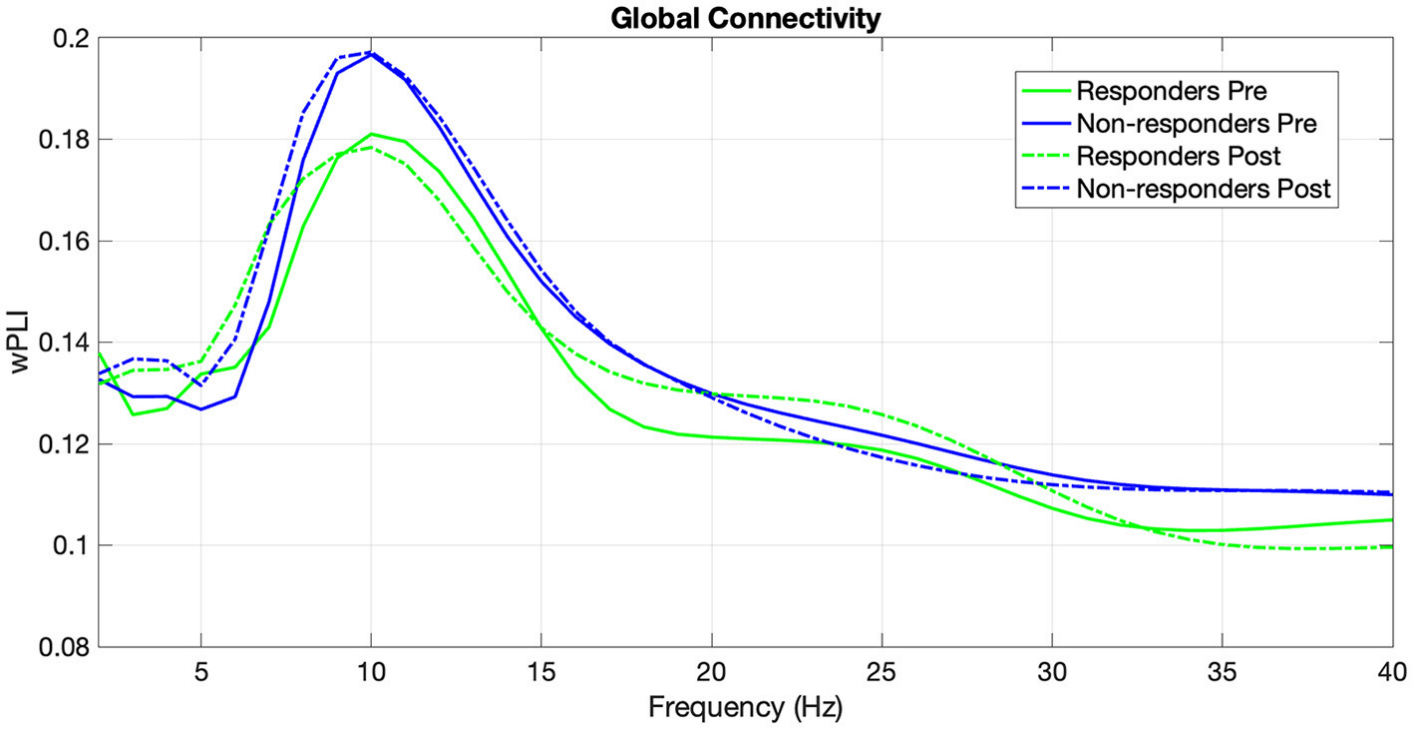
Weighted phase-lag index of responders and non-responders in the frequency range from 2 to 40 Hz at pre-treatment and post-treatment time points.

We computed the wPLI (Vinck et al., 2011) of responders and non-responders at both time points (pre- and post-treatment) from 2 to 40 Hz in 1 Hz steps (Figure 4).

For the theta band (4–7 Hz), the between-subjects analysis revealed no significant main effect of Group *F*_(1, 83)_ = 0.0941, *p* = 0.760. The main effect of Time (pre-post) was significant, *F*_(1, 83)_ = 7.9575, *p* = 0.006, with higher global wPLI in the post-intervention session. However, the interaction effect between Time and Group was not statistically significant, *F*_(1, 83)_ = 0.0322, *p* = 0.858.

For the alpha band (8–12 Hz), although mean alpha connectivity values were higher for the non-responders Group, the analysis revealed no significant main effect of Group *F*_(1, 83)_ = 0.923, *p* = 0.339. The main effect of Time was not significant, *F*_(1, 83)_ = 0.0247, *p* = 0.876. Similarly, the interaction effect between Time and Group was not statistically significant, *F*_(1, 83)_ = 0.0469, *p* = 0.829.

For the beta band (13–30Hz), the between-subjects analysis revealed no significant main effect of Group *F*_(1, 83)_ = 0.897, *p* = 0.346. The main effect of Time was not significant, *F*_(1, 83)_ = 0.0114, *p* = 0.915. Similarly, the interaction effect between Time and Group was not statistically significant, *F*_(1, 83)_ = 0.0770, *p* = 0.782.

## 4 Discussion

In the present study, we performed a randomized, double-blind and sham-controlled clinical trial in order to contribute to a better understanding of the neural mechanisms underlying chronic pain relief after tES. The first objective was to explore how oscillatory activity in chronic pain patients is modulated by 15 sessions of home-based tDCS or tACS. The second objective was to examine the pattern of oscillatory activity of chronic pain patients who respond positively to tES treatment.

With the protocols used in this study, we did not find evidence that oscillatory activity in chronic pain patients (measured with mean PSD) was modulated following a 15-day home-based intervention of active tDCS or tACS compared to sham stimulation. However, we found that patients who reported pain relief after a tES treatment (i.e., responders) had a distinct neural signature, with higher activity in the theta frequency band, compared to non-responders. No changes in mean PSD in other frequency bands were found between responders and non-responders, and neither changes in global connectivity in the theta, alpha and beta frequency bands.

Although differences in oscillatory activity have been observed in chronic pain patients compared to healthy populations (see Pinheiro et al., 2016; Ploner et al., 2017 for a review), little has been studied about how pain relief treatment might affect oscillatory activity at rest. In a recent review, Zebhauser et al. (2023) found no clear association between pain relief and changes in oscillatory activity following different kinds of treatment. The lack of research is even more evident regarding tES treatment, as very few studies have approached this topic. Results from those studies showed an increase in alpha oscillations at rest after applying tDCS (De Melo et al., 2020; Thibaut et al., 2017) or tACS (Ahn et al., 2019; Bernardi et al., 2021). We have not been able to replicate these results with our current protocols for tDCS and tACS.

This discrepancy in results could be explained by the differences in the stimulation protocols used for each study. There is substantial heterogeneity in the tDCS and tACS protocols used throughout the literature for pain relief in chronic pain, with studies differing in the number of sessions, stimulation duration and parameters, electrode placement, etc. This lack of standardized protocols in the field is also evident when analyzing the effects of tDCS and tACS in brain activity at rest, as there are only two previous studies for each technique, which differ in some aspects from the protocols used in the present study. In this regard, the previous studies recorded post-treatment rsEEG immediately after delivering the last tES session; while in our study, as we used a home-based protocol for tES, post-treatment rsEEG had to be recorded at least 24 h after the last stimulation session. It is unclear how long the after-effects of tDCS and tACS in brain oscillatory activity at rest might last. Therefore, it is possible that after-effects in brain oscillatory activity might last less than 24 h for both techniques, which meant that, at the time of our recordings, the possible modulation of oscillatory activity might have disappeared already.

Moreover, although the present study counts with a larger sample size of patients, it includes patients diagnosed with different conditions that caused chronic pain, while the aforementioned studies have focused on one chronic pain condition (e.g., fibromyalgia, low back pain, and chronic visceral pain, respectively). Although pain chronification might share common neural mechanisms for different disorders, it could be possible that brain oscillatory activity at rest might differ depending on diagnosis or on the time elapsed since pain became chronic. In fact, some studies have found decreased theta activity at rest in fibromyalgia patients (Hargrove et al., 2010; Navarro López et al., 2015), that contrast with the common finding of increased theta activity in other chronic pain conditions (see a review in Pinheiro et al., 2016; Ploner et al., 2017; Silva-Passadouro et al., 2024). Besides, years living with chronic pain have been associated to changes in the Default Mode Network (DMN) activity (Heukamp et al., 2025). These idiosyncrasies in oscillatory brain activity at rest of different chronic pain conditions as well as the variability in the time course of chronic pain, might, in part, explain our results despite having a larger sample size.

Interestingly, we found higher theta activity in patients who obtained pain relief with tES (either tACS or tDCS -responders-) compared to patients who did not respond to the treatment (non-responders). To our knowledge, this is the first study to explore the oscillatory brain activity and functional connectivity of responders to tES.

Not all chronic pain patients respond to tDCS and tACS, and these findings raise the question of whether brain activity at rest might influence whether a patient might benefit from a specific tES protocol. Previous studies have shown different brain activity at rest for patients that respond to tDCS treatment for tinnitus (Vanneste et al., 2011), post-traumatic stress disorder (Kim et al., 2022) or depression (Albizu et al., 2024). Furthermore, a recent study with tACS in fibromyalgia patients has shown success tailoring the frequency of the tACS received according to the pre-intervention EEG characteristics of the patients (compared to the EEG of healthy controls). That way, delivering beta-tACS (30 Hz) to patients with lower amplitudes of slow frequencies at rest, and theta-tACS (4 Hz) to patients with lower amplitudes of high frequencies at rest, combined with rehabilitation, reduced pain and improved quality of life (Bernardi et al., 2021).

In addition, changes in theta oscillations at rest have been consistently associated with chronic pain. An overactivation of theta activity has been reported in areas associated to pain processing, such as the inferior parietal cortex and the primary, secondary and supplementary somatosensory cortices (Stern et al., 2006). Furthermore, increases in this activity are commonly observed in patients with neurogenic pain and migraines (Di Pietro et al., 2018; Prichep et al., 2018; Sarnthein et al., 2006; Vuckovic et al., 2014; for a review, see Mussigmann et al., 2022), and a recent study found that increased parietal theta activity was associated with changes in pain intensity (Barbosa et al., 2024). The result that patients with higher theta activity specially benefited from active tES opens a new avenue for personalizing neurostimulation interventions. Nevertheless, it should be replicated in further studies, using a prospective validation design in a large cohort of patients.

Differences in functional connectivity have been described in chronic pain populations when compared to healthy populations, with one of the most consistent findings in fMRI studies being changes in the DMN of chronic pain patients (Thorp et al., 2018). Furthermore, decreased global connectivity in the theta frequency has been associated with pain intensity after chronic pain treatment (Heitmann et al., 2022), and its decrease has been described in fibromyalgia patients compared to healthy population (Choe et al., 2018). However, we have been unable to find changes in global connectivity that could characterize responders and non-responders to tES treatment in the theta, alpha or beta frequency bands. Other studies have found changes in connectivity of chronic pain patients compared to healthy population between the anterior cingulate cortex and the somatosensory cortex (Vanneste and De Ridder, 2021), or in connectivity in the prefrontal cortex (Ta Dinh et al., 2019).

More research is needed to better characterize the brain activity and connectivity of responders and non-responders to tES in different populations with chronic pain, and to evaluate the effect of time since the pain became chronic on brain activity. Future research should look more in depth into these variances in theta activity at rest, also taking into account how different tES protocols might interact with these particularities in theta activity to bring pain relief. Related to this, another topic worth investigating for future studies is the possible individualization of tDCS and tACS protocols to tailor stimulation parameters to the particular alterations observed in the brain activity of patients.

As with any scientific study, the present study has its share of limitations that should be taken into account. First, as mentioned above, due to the format for the stimulation delivery (at home), EEG activity post-treatment could not be recorded immediately following the last stimulation session, in turn being recorded with a minimum of 24 h delay. This could have resulted in the attenuation (or outright disappearance) of possible after-effects of tDCS and tACS in brain oscillatory activity at rest. More research is needed into the long-term duration of tDCS and tACS after-effects in oscillatory activity at rest. A common limitation in tES studies for chronic pain is small sample sizes. In the present study we were able to recruit a large sample of over 100 patients, but heterogeneity of chronic pain conditions and time elapsed since the diagnoses could have influenced the obtained results. Furthermore, the sample in the present study comprised a larger number of women than men; which could limit the generalization of the obtained results to the male population. Besides, the heterogeneity of pharmacological treatments followed by the participants could have interacted with the stimulation intervention. It has been reported that some medications might interact with tDCS protocols (McLaren et al., 2018), but it is unknown if such interactions exist with tACS. Although we required that treatment remained unchanged during the study, there is inevitable inter-individual variance in pharmacological treatment that could have influenced the study outcomes.

### 4.1 Conclusion

In summary, we found that chronic pain patients who report pain relief following a home-based treatment of tDCS or tACS show distinct neural activity than non-responders to the same tES interventions. This consisted of a higher theta oscillatory activity at rest, especially in temporal, parietal and central electrode sites. To our knowledge, this is the first study reporting on a distinct neural signature in chronic pain patients who benefit from tES in comparison with those who do not. In light of these findings, personalized tES treatment adjusted to the brain activity of chronic pain patients might be a promising strategy for non-invasive and accessible pain relief.

## Data Availability

The datasets presented in this article are available upon request, with the condition that compliance with ethical procedures governing the reuse of the data must be maintained.

## Footnotes

^1^In this paper we only analyzed pain intensity NRS, but other measures and instruments were also included in the trial. See pre-registration at clinicaltrials.gov (ID number: NCT05099406).
